# Acute fluid shifts influence the assessment of serum vitamin D status in critically ill patients

**DOI:** 10.1186/cc9341

**Published:** 2010-11-26

**Authors:** Anand Krishnan, Judith Ochola, Julie Mundy, Mark Jones, Peter Kruger, Emma Duncan, Bala Venkatesh

**Affiliations:** 1 Intensive Care Unit, Princess Alexandra Hospital, University of Queensland, Ipswich Road, Woolloongabba, QLD 4102, Australia; 2 Department of Cardiothoracic Surgery, Princess Alexandra Hospital, University of Queensland, Ipswich Road, Woolloongabba, QLD 4102, Australia; 3 Department of Diabetes and Endocrinology, Royal Brisbane Hospital, University of Queensland, Bowen Bridge Road, Herston QLD 4029, Australia; 4 Department of Intensive Care, The Wesley Hospital, 451 Coronation Drive, Auchenflower, QLD 4066, Australia

## Abstract

**Introduction:**

Recent reports have highlighted the prevalence of vitamin D deficiency and suggested an association with excess mortality in critically ill patients. Serum vitamin D concentrations in these studies were measured following resuscitation. It is unclear whether aggressive fluid resuscitation independently influences serum vitamin D.

**Methods:**

Nineteen patients undergoing cardiopulmonary bypass were studied. Serum 25(OH)D_3_, 1α,25(OH)_2_D_3_, parathyroid hormone, C-reactive protein (CRP), and ionised calcium were measured at five defined timepoints: T1 - baseline, T2 - 5 minutes after onset of cardiopulmonary bypass (CPB) (time of maximal fluid effect), T3 - on return to the intensive care unit, T4 - 24 hrs after surgery and T5 - 5 days after surgery. Linear mixed models were used to compare measures at T2-T5 with baseline measures.

**Results:**

Acute fluid loading resulted in a 35% reduction in 25(OH)D_3 _(59 ± 16 to 38 ± 14 nmol/L, *P *< 0.0001) and a 45% reduction in 1α,25(OH)_2_D_3 _(99 ± 40 to 54 ± 22 pmol/L *P *< 0.0001) and i(Ca) (*P *< 0.01), with elevation in parathyroid hormone (*P *< 0.0001). Serum 25(OH)D_3 _returned to baseline only at T5 while 1α,25(OH)_2_D_3 _demonstrated an overshoot above baseline at T5 (*P *< 0.0001). There was a delayed rise in CRP at T4 and T5; this was not associated with a reduction in vitamin D levels at these time points.

**Conclusions:**

Hemodilution significantly lowers serum 25(OH)D_3 _and 1α,25(OH)_2_D_3_, which may take up to 24 hours to resolve. Moreover, delayed overshoot of 1α,25(OH)_2_D_3 _needs consideration. We urge caution in interpreting serum vitamin D in critically ill patients in the context of major resuscitation, and would advocate repeating the measurement once the effects of the resuscitation have abated.

## Introduction

Vitamin D is synthesised in the skin through UV action on 7-dehydrocholesterol, to cholecalciferol. It is transported in the blood by the Vitamin D binding protein (VDBP) to the liver where it undergoes 25 hydroxylation to form 25(OH)D_3, _which in turn undergoes 1α hydroxylation (especially, but not exclusively in the kidneys) to form 1α,25(OH)_2_D_3_. Its traditionally recognised role is to maintain adequate serum calcium and phosphate levels, for bone mineralisation and optimal cardiac [1] and skeletal muscle function [2]. However, increasing data from biochemical, and molecular genetic studies indicate that vitamin D has a much wider range of actions, which are termed pleiotropic effects. These include potentiation of antimicrobial action, and cardioprotective and immunomodulatory effects [3]. The immunomodulatory properties of vitamin D have been shown to improve outcomes in transplant recipients [4], reduce relapses in multiple sclerosis [5], and may reduce the development of type I diabetes mellitus [6]. In the general population there is a 26% increase in all-cause mortality in those in the lowest quartile of 25 (OH)D_3 _levels when compared to the highest quartile [7].

Awareness of the pleiotropic effects of Vitamin D has captured the interest of intensivists. Critically ill patients with prolonged stays in an intensive care unit may develop vitamin D deficiency for a number of reasons, including lack of exposure to sunlight, malnutrition, decreased renal 1α hydroxylation and increased tissue conversion of 25(OH)D_3 _to 1α,25(OH)_2_D_3 _during acute stress and the inflammatory response [8,9]. An additional contributor to vitamin D deficiency in critically ill patients may be perturbations in serum albumin and VDBP. Reductions in serum concentrations of these proteins will influence total circulating concentrations of vitamin D [10]. Published data suggest a significantly higher incidence of vitamin D deficiency and bone resorption in chronically critically ill patients [11]. Van den Berghe *et al*. [9] showed the levels of both 25(OH)D_3 _and 1α,25(OH)_2 _D_3 _are low on admission to ICU compared to age-matched controls. Evidence from a recent case series [12] demonstrated significantly worse outcomes for patients with reduced serum levels of 25(OH)D_3 _in critical illness, although a direct causal effect has not been proven. All this has generated renewed interest in the pharmacodynamics of vitamin D, especially in the critically ill patient. This together with accumulating evidence on hypovitaminosis D in the critically ill has prompted calls for supplementation in these patients.

However, there are several limitations to the published data. It is frequently unclear as to when the "baseline" measurements were performed. Critically ill patients on admission to the hospital or intensive care unit often receive large volumes of intravenous fluids to correct hypovolemia and hypotension, and the extent of volume replacement is often directly related to the severity of acute illness [13]. Acute expansion of the intravascular volume is associated with reduction in levels of various electrolytes, proteins and blood components due to hemodilution [14]. Whether the post dilution effect would be responsible for the observed low baseline levels of 25(OH)D_3 _needs investigation. Moreover, critically ill patients are often "waterlogged" and are slow in clearing body water. Consequently any dilutional effect of baseline resuscitation may have an impact on plasma concentrations beyond the resuscitation period. How this will influence the interpretation of Vitamin D in the peri-resuscitation period remains unclear. Finally, most studies have examined 25(OH)D_3 _, while the active hormone is 1α,25(OH)_2_D_3_. Whether changes in 1α,25(OH)_2_D_3 _parallel those of 25(OH)D_3_, during volume loading and clearance also remain unknown. Of note, the water-solubility and half-lives of these different forms of vitamin D are quite different [15].

We chose cardiopulmonary bypass as a clinical model to test this question. Patients undergoing routine elective cardiac surgery are not acutely unwell, and receive a standard volume fluid load when going on to cardiopulmonary bypass (CPB) which is cleared over the next several days. They usually have a predictable course of recovery and hence provide an ideal model to look at the effects of acute fluid loading on vitamin D levels.

## Materials and methods

This study was conducted at the Princess Alexandra Hospital in Brisbane, a tertiary-care referral centre with one of the biggest cardiac surgical services in Australia. The study was approved by the institution's Human Research Ethics Committee and written, informed consent was obtained from patients prior to inclusion.

### Inclusion criteria

Patients aged 18 years or older scheduled to undergo elective cardiac surgery under cardiopulmonary bypass were eligible for enrolment into the study.

Patients were excluded from the study if they met any of the following criteria: 1) if they were undergoing urgent cardiac surgery, 2) if they had chronic hepatic dysfunction (grade greater than Child-Pugh A), 3) if they had renal dysfunction (creatinine >200 micromol/L).

The conduct of anaesthesia and surgery was as per standard practice. Briefly, the cardiopulmonary bypass (CPB) circuit incorporated a membrane oxygenator (Dideco Avante, Cellplex, Sorin, Mirondola, Italy) and heart lung machine (Jostra HL 20, Maquet Critical Care AB, Solna, Sweden). Pump rate was set at 2.4 L/minute/m^2 ^and temperature ranged from 32 to 35°C. The circuit was primed with 2 L of Plasma-Lyte 148 (Na^+ ^140 mmol/L, Cl^- ^98 mmol/L, K^+ ^5 mmol/L, Mg^++ ^1.5 mmol/L, Acetate 27 mmol/L, Gluconate 23 mmol/L), a commercially available balanced crystalloid solution. (Baxter Healthcare, Toongabbie, NSW, Australia).

Following surgery all patients were admitted to the intensive care unit for 24 hours. They were all ventilated postoperatively as per standard protocol. Vasoactive drugs and external cardiac pacing were commenced according to clinical need. In addition, all patients received stress ulcer prophylaxis, analgesia, and anticoagulation prophylaxis with subcutaneous heparin and aspirin routinely commenced the morning after their operation. Patients were discharged from the ICU after 24 hours and from the hospital between Day 5 and Day 7 postoperatively.

Fluid intake and output were recorded from the commencement of surgery till discharge from the hospital; these were done relative to a baseline value of zero litres just prior to commencement of surgery. Patients were weighed at baseline, 24 hours after surgery and on Day 5.

### Serum measurements

Blood was sampled at the following time points:

T1) Immediately before commencing cardio-pulmonary bypass; T2) Five minutes after commencement of bypass, prior to placement of the aortic cross-clamp (immediately after a large increase in blood volume due to the mixture with the CPB circuit prime); T3) On return to the intensive care unit after surgery, T4) 24 hours after surgery, T5) 5 days after surgery.

At each time point serum 25(OH)D_3_, 1 α,25(OH)_2_D_3_, parathyroid hormone (PTH), total and ionised calcium, total magnesium and phosphate and C-reactive protein were measured.

### Assays

Serum 25(OH)D_3 _and 1α,25(OH)_2_D_3 _were measured by LC MSMS system (Waters Corp Milford, MA, USA) (between-run coefficient of variations (CV%): vitamin D2 at 42.0 nmol/L 5.9%, and at 91.0 nmol/L 7.7%; vitamin D3 at 76.0 nmol/L 15.3%, and at 181 nmol/L 6.8%). Parathormone estimation was an immunoassay performed on Siemens Immulite analyser (Siemens Healthcare Diagnostics, Medfield, MA, USA) with antibodies specific for the C-terminal region thereby recognising only intact PTH (between-run CV%: at 1.18 pmol/L 8.8%; at 6.69 pmol/L 5.3%, and at 42.87 pmol/L 5.0%, The total calcium (Ca), magnesium (Mg^2+^) and inorganic phosphorus (PO_4_^3-^) were measured on Beckman DxC 800 general chemistry analysers (Beckman Coulter Diagnostics, Fullerton, CA, USA) by ion selective electrode, and photometric methods (between-run precision limits: Ca at 1.98 mmol/L 1.7% and at 2.96 mmol/L 1.7%; Mg^2+ ^at 0.75 mmol/L 3.8% and at 1.60 mmol/L 2.4%; PO_4_^3- ^at 0.98 mmol/L 5.1% and at 3.19 mmol/L 5.1%). The i(Ca) was measured on Siemens Rapidlab 1265 blood gas analysers (Siemens Healthcare Diagnostics, Medfield, MA, USA) by ion selective electrode (between-run CVs: at 0.80 mmol/L 1.8% and at 1.60 mmol/L 1.5%). CRP was measured on Beckman DxC800 general chemistry analysers using a turbidimetric method (between-run CVs: at 4.9 mg/L 5.3% and at 10.5 mg/L 3.6%).

### Statistical methods

The data were analysed using SAS version 9.2 for Windows (SAS Institute, Cary, NC, USA). Linear mixed models were used to compare the measures of vitamin D, electrolytes and CRP taken at time-points 2, 3, 4 and 5 with measures taken at baseline (time-point 1). In separate analyses, the vitamin D measures were specified as dependent variables in linear mixed models and then tested for association with CRP, fluid balance and electrolytes. R-square was used to express the magnitude of correlation between the vitamin D measures and the other parameters [16].

## Results

Nineteen patients were included in the study. All patients successfully underwent cardiac surgery and were discharged live from the hospital. The baseline, demographic and operative data are summarised in Table 1.

**Table 1:** Demographic data

Total number of patients	19
Male/female distribution	14 M, 5 F
Mean age (yrs)	59 ± 12
Types of operations	7 CABG, 11 valvular procedures and 1 CABG + valve
Mean baseline creatinine (micromol/L)	80 ± 22
Mean Baseline weight (kg)	88 ± 21
Mean BMI (kg/m^2^)	30 ± 6
Mean bypass time (min)	108 ± 49
Mean cross clamp time (min)	78 ± 45

BMI, body mass index; CABG, coronary artery bypass grafts.

### Changes in fluid balance and body weight

Baseline values were taken as zero fluid balance status. Predictably, there were positive fluid balances at T2 (3.5 ± 1.2 L), T3 (3.0 ± 1.5 L), and T4 (2.5 ± 1.2 L). On Day 5, the fluid balance had started to return towards baseline (1.1 ± 0.8 L). Changes in bodyweight followed fluid balance profile. The mean bodyweight at baseline was 88 ± 20 kg, increased to 91 ± 22 kg at T4 and returned to 87 ± 21 kg at T5. All patients received at least one dose of frusemide as part of routine postoperative care. None of the patients received blood transfusions during CPB.

### Serum Vitamin D (25(OH)D_3 _and 1α,25(OH)_2_D_3_)

The mean baseline, serum 25(OH)D_3 _was 59 ± 16 nmol/L. At T2, (immediately after mixture with the pump prime), there was a 35% reduction in 25(OH)D_3 _to 38 ± 14 nmol/L (*P *< 0.0001). Serum 25(OH)D_3 _continued to remain low and returned to baseline only on Day 5 (T5).

**Figure 1:**
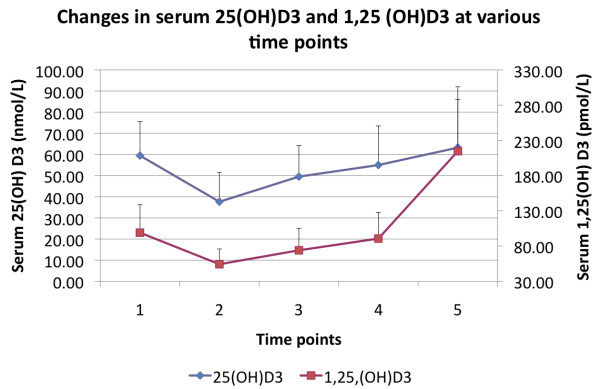
**An illustration of the changes in serum 25(OH)D_3 _(filled diamonds) and 1α,25(OH)_2_D_3 _(filled squares) concentrations across the five time points**.

Serum 1α,25(OH)_2_D_3 _appeared to follow a similar temporal course to that of 25(OH)D_3 _initially. The mean baseline concentrations were 99 ± 40 pmol/L Hemodilution resulted in significant reductions (45%) in 1,25(OH)_2_D_3 _to 54 ± 22 pmol/L (*P *< 0.0001). In contrast to 25(OH)D_3_, concentrations of 1 α,25(OH)_2_D_3 _demonstrated a significant overshoot above baseline on Day 5 (T5) to 214 ± 91 pmol/L (*P *< 0.0001). These are illustrated graphically in Figure 1.

### Serum PTH, calcium, magnesium and phosphate

**Figure 2:**
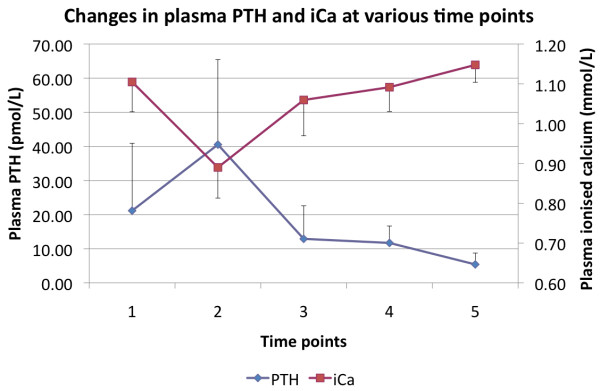
**An illustration of the changes in serum PTH (filled diamonds) and ionised calcium (filled squares) concentrations across the five time points**.

Changes in serum PTH appeared to follow an opposite course to that of 25(OH)D_3 _and 1α,25(OH)_2_D_3_. Baseline levels (21 ± 19 pmol/L) were elevated, rose significantly with hemodilution to 41 ± 25 pmol/L (*P *< 0.0001) and returned to baseline at 24 hours and levels were significantly low (5 ± 3 pmol/L) on Day 5. Changes in serum ionised calcium, magnesium and phosphate are shown in Table 2 and those of ionised calcium and PTH are shown in Figure 2.

**Table 2:** Changes in serum total and ionised calcium, magnesium and phosphate

	T1	T2	T3	T4	T5
Total calcium (mmol/L)	2.2 ± 0.1	1.9 ± 0.1*	2.1 ± 0.2*	2.1 ± 0.1*	2.3 ± 0.1*
i[Ca] (mmol/L)	1.1 ± 0.1	0.9 ± 0.1*	1.1 ± 0.1	1.1 ± 0.1	1.2 ± 0.04
Mg^++ ^(mmol/L)	1.1 ± 0.4	1.3 ± 0.4	1.3 ± 0.2	1.1 ± 0.2	0.9 ± 0.1*
PO4^3- ^(mmol/L)	1.1 ± 0.2	0.9 ± 0.2*	1.0 ± 0.2*	1.2 ± 0.3	1.1 ± 0.3

*Significantly different from baseline (*P *< 0.01).T1 - baseline, T2 - five minutes after onset of cardiopulmonary bypass (CPB) (time of maximal fluid effect), T3 - on return to the intensive care unit, T4 - 24 hrs after surgery, T5 - five days after surgery.

### Serum CRP

Serum CRP levels were within the normal range at baseline (6 ± 9 mg/L). There was a statistically significant (but clinically insignificant) drop with hemodilution (4 ± 5 mg/L, *P *= 0.04). At 24 hours (T4) significant elevations in CRP with respect to baseline were noticed (82 ± 40 mg/L, *P *< 0.0001), and remained persistently elevated even on Day 5 (134 ± 58 mg/L, *P *< 0.01).

### Serum albumin and creatinine

The mean baseline serum albumin was 34 ± 4 g/L. At T2, the concentrations fell by 30% to 24 ± 4 g/L (*P *< 0.0001), followed by a gradual return towards baseline over the following time points: T3 to 30 ± 4 G/L (*P *< 0.001), T4 to 32 ± 4 G/L (*P *= 0.11) and T5 32 ± 3 g/L (*P *= 0.06).

The mean baseline creatinine was 81 ± 22 μmol/L (normal reference range <90 μmol/L), which fell to 71 ± 16 μmol/L at T2 (*P = *0.002). There was both a clinically and a statistically insignificant overshoot from baseline at T3, T4 and T5: 89 ± 38 μmol/L (*P *= 0.23), 90 ± 40 μmol/L (*P *= 0.15) and 90 ± 43 μmol/L respectively (*P *= 0.2).

### Effect of different variables on vitamin D

Using 25(OH)D_3 _as a dependent variable, linear mixed models were used and tested for independent association of changes in 25(OH)D_3 _with fluid balance, CRP and electrolytes. Fluid balance showed a signifcant negative association with both 25(OH)D_3 _(effect size -4.9, CI -6.4 to -3.4, *P *< 0.0001) and 1 α,25(OH)_2_D_3 _(effect size -14.0, CI -22 to -6, *P *< 0.001). Changes in serum albumin were strongly correlated with 25(OH)D_3 _(*P *< 0.0001) and 1α,25(OH)_2_D_3 _(*P *< 0.0002). Changes in serum creatinine were correlated with 25(OH)D_3_, (*P *< 0.05), but not 1α,25(OH)_2_D_3 _(*P *= 0.94).

Serum CRP showed a significant positive association with both 25(OH)D_3 _(effect size 0.08, 0.02 to 0.14, *P *< 0.01) and 1 α,25(OH)_2_D_3 _(effect size 0.62, CI 0.39 to 0.84), *P *< 0.0001).

Ionised calcium was strongly associated with both 25(OH)D_3 _(effect size 67.2 (CI 47.6 to 87, *P *< 0.0001) and 1 α,25(OH)_2_D_3 _(effect size 249 (CI 127 to 371, *P *< 0.0001). Total calcium was also strongly associated with both 25(OH)D_3 _(*P *< 0.0001) and 1 α,25(OH)_2_D_3 _(*P *< 0.0001). Phosphate concentrations did not show any significant association with both 25(OH)D_3 _and 1 α,25(OH)_2_D_3 _concentrations. Magnesium was less strongly associated with 25(OH)D_3 _than calcium (*P *< 0.05).

There was no association between changes in fluid balance and CRP (effect size -4.1, CI -11.3 to 3.1, *P *= 0.26).

### Classification of patient's vitamin D status at various time points

Table 3 illustrates the number of patients who would have been classified as vitamin D insufficient or deficient at various time points based on cut offs of 25(OH)D_3 _of 60 nmol/L and 30 nmol/L, respectively.

**Table 3:** Proportion of patients who would have been classified as Vitamin D insufficient or deficient at various time points

	T1	T2	T3	T4	T5
Vitamin D insufficiency (25(OH)D_3_ <60 nmol/L)	47%	89%*	68%	53%	47%
Vitamin D deficiency (25(OH)D_3_ <30 nmol/L)	5%	37%**	11%	11%	6%

*P < 0.01, significantly different from baseline

**P < 0.05, significantly different from baseline

T1 - baseline, T2 - five minutes after onset of cardiopulmonary bypass (CPB) (time of maximal fluid effect), T3 - on return to the intensive care unit, T4 - 24 hrs after surgery, T5 - five days after surgery.

The individual patient's endocrine data, calcium and albumin concentrations at various time points with changes in fluid balance at each of those time points are supplied in a table form and available in Additional file 1.

## Discussion

The cardinal findings of the study are that intravascular volume loading significantly affects both 25(OH)D_3 _and 1α,25(OH)_2_D_3_. Changes in 25(OH)D_3 _and 1α,25(OH)_2_D_3 _were accompanied by reciprocal alterations in PTH concentrations. Both volume shifts and inflammatory response appeared to have independent effects on both 25(OH)D_3 _and 1α,25(OH)_2_D_3 _concentrations.

### Changes in serum Vitamin D and PTH

Volume loading clearly appeared to impact on 25(OH)D_3 _and 1α,25(OH)_2_D_3 _. At baseline, 25(OH)D_3_, was low in keeping with the reported prevalence of hypovitaminosis D in the community.

On initiation of CPB, there was an increase in the circulating blood volume by a volume of 2 L owing to the obligatory addition of the pump prime. Assuming a normal blood volume of 5 L, this prime would increase the blood volume by an additional 40%. Therefore, reductions of 25(OH)D_3_, and 1α,25(OH)_2_D_3 _of 35% and 45% respectively at T2 would be consistent with this dilution effect. Albumin binds to Vitamin D and, therefore, reductions in albumin will be accompanied by parallel reductions in the latter. Further support for this haemodilution effect is shown by the strong correlation between changes in serum albumin and vitamin D. Other possible explanations include adsorption of vitamin D by the plastic tubing and catabolism of these hormones through 24-hydroxylation to calcitroic acid [17]. Another possibility to consider is a reduction in serum VDBP concentrations from dilution as well adsorption to plastic as it is a protein and, therefore, may carry an electrical charge. However, the strong temporal relationship of serum concentrations to hemodilution, the rapidity of the drop together with a subsequent rise with fluid clearance and the magnitude of the drop being explained by the proportional rise in circulating volume argue in favour of a dilution effect as the preponderant cause. Plasma concentrations of these hormones started to rise by T3. This is likely due to intravascular volume losses owing to separation from CPB, surgical losses and diuresis. Values only reached baseline by 24 hours (T4) in the case of 1α,25(OH)_2_D_3_, while statistically significant differences were still noticeable for 25(OH)D_3 _at this time point.

At T5, 25(OH)D_3 _returned to baseline, while 1α,25(OH)_2_D_3 _demonstrated a statistically significant overshoot from baseline. The mechanism of the delayed rise in 1α,25(OH)_2_D_3 _is unclear. The most likely explanation is the induction of 1- α hydroxylase by PTH. The rise in PTH at T2 may be significant. PTH-dependent synthesis of new 1- α hydroxylase takes several hours and it is therefore likely that the initial rise in PTH was accompanied by a delayed rise in 1α,25(OH)_2_D_3 _[18].

Moreover, there was also evidence of inflammation as evidenced by a delayed rise in CRP. Macrophages are potent sources of 1- α hydroxylase and may also have contributed to the elevated 1α,25(OH)_2_D_3 _levels through extra renal production of 1α,25(OH)_2_D_3 _[19]. Alterations in VDBP are known to influence monocyte responses to 25(OH)_2_D_3 _and 1α,25(OH)_2_D_3 _[20]. Whether it played a role in the delayed rise in 1α,25(OH)_2_D_3 _remains speculative. Support for the extra-renal contribution to the delayed rise in 1α,25(OH)_2_D_3 _also comes from the lack of a strong correlation between creatinine levels and 1α,25(OH)_2_D_3_. Other more chronic causes appear unlikely as the values were normal at baseline only five days prior.

We also found that PTH levels further rose significantly after commencement of CPB. The reduction in ionised calcium as a result of hemodilution and chelation by acetate-containing fluid used in priming the CPB may be responsible for the rapid rise in PTH, as part of the normal physiological response to hypocalcaemia. However, patients also had a significant and abrupt increase in serum magnesium which acutely increases PTH secretion. It is not possible in this model to distinguish between these stimuli for PTH secretion.

### Clinical significance of our findings

To our knowledge this is the first study to examine the effects of volume loading on serum vitamin D concentrations. Our data clearly demonstrate the impact of fluid loading on serum vitamin D. Over the past decade, some reports have highlighted the prevalence of vitamin D deficiency and suggested an association with excess mortality in critically ill patients [9,12]. Recently, Lucidarme *et al*. identified a high prevalence of 25(OH)D_3 _insufficiency and deficiency in a prospective observational study of 134 critically ill patients [21]. They identified albumin, spring admission and SAPS II score as predictors of hypovitaminosis but levels of 1α,25(OH)_2_D_3 _were not estimated. More importantly, in none of the above studies have the authors specified the timing of blood sample collection in relationship to fluid resuscitation. It is, therefore, unclear whether aggressive fluid resuscitation may have influenced serum vitamin D concentrations. In Table 3 the data clearly illustrate the potential for misclassification of patients as Vitamin D insufficient or deficient purely from a fluid effect. Moreover, critically ill patients are often water logged and are unable to clear a fluid load owing to hypoalbuminemia, renal dysfunction, and high ADH levels. Consequently any effects of a fluid load may be longstanding. In patients with severe baseline deficiency such as 25(OH)D_3 _values <15 nmol/L, it is likely that fluid loading may not have a significant physiological impact. The distribution space of 25(OH)D_3 _is similar to that of plasma, while 1α,25(OH)_2_D_3 _is closer to that of intracellular water. Consequently the tonicity of the diluting fluid and the resultant expansion of the intravascular and the interstitial space become relevant. Based on our data, we would urge caution in interpreting serum vitamin D in critically ill patients in the context of major resuscitation and would advocate repeating the measurement once the effects of the resuscitation have abated. While not examined in this study, another potential source of error in assessing vitamin D status is the assay variability as differences in vitamin D concentrations have been reported on the same sample depending on the methodology used [22].

Inflammatory response has been suggested to reduce Vitamin D concentrations [9]. In this study, biochemical evidence of inflammatory response began to emerge only 24 hours after bypass in keeping with previously published data; however this was accompanied by a rise in 1α,25(OH)_2_D_3 _and there was a strong association between CRP and 25(OH)D_3 _and 1α,25(OH)_2_D_3_.

### Limitations of the study

Although the study is limited by the small sample size, the effect size was large enough to produce significant results. As stated earlier, the model of CPB was chosen because, patients undergoing routine elective cardiac surgery are not acutely unwell, hemodilution is an integral aspect of cardiopulmonary bypass (CPB) which is cleared subsequently and these patients usually, have a predictable course of recovery. Although the lower levels of patient acuity and minimal organ dysfunction raise the question whether these results can be transposed to other groups of critically ill patients, we assert that acute physiological effects of hemodilution are likely to be comparable across various patient groups, although the magnitude of the effect may be variable. As the protocol did not incorporate measurements between Day 1 and Day 5, it was not possible to elucidate the mechanism behind delayed changes in vitamin D concentrations. The measurement of VDBP levels would have provided useful information in terms of understanding of the mechanisms. CPB also initiates an inflammatory response; however, this is often a delayed process manifesting 12 to 24 hours after bypass (as observed in this study), whilst the major volume changes occurred soon after commencement of CPB. The early changes are therefore likely to be due to volume effects. Moreover, analysis of our data also showed that fluid shifts correlated with changes in vitamin D but not CRP.

## Conclusions

In conclusion, hemodilution and acute fluid shifts significantly lower serum 25(OH)D_3_ and 1α,25(OH)_2_D_3. _These may take up to 24 hours to resolve. The magnitude of changes in 25(OH)D_3 _and 1α,25(OH)_2_D_3 _appeared to be proportional to the extent of volume change. Based on our data, we would urge caution in interpreting serum vitamin D in critically ill patients in the context of major resuscitation and would advocate repeating the measurement once the effects of resuscitation have abated.

## Key messages

• Acute fluid loading during critical illness markedly reduces serum 25(OH)D_3 _and 1α,25(OH)_2_D_3 _concentrations.

• Serum concentrations may take up to 24 hours to return to baseline.

• We urge caution in interpreting serum vitamin D in critically ill patients in the context of major resuscitation and would advocate repeating the measurements once the effects of resuscitation have abated.

## Abbreviations

CPB: Cardiopulmonary bypass; CRP: C-reactive protein; CA^2+^: serum total calcium; [CA^2+^]: Serum ionised calcium; MG^2+^: serum magnesium; (PO_4_^3-^): Serum inorganic phosphorus; PTH: Parathormone; 25(OH)D_3_: 25-hydroxy vitamin D3; 1α,25(OH)_2_D_3_: 1,25-dihydroxy vitamin D3.

## Competing interests

The authors declare that they have no competing interests.

## Authors' contributions

AK was involved in study design, data analysis and manuscript preparations. JO was involved in study design, sample collection and storage and data analysis. JM was involved in study design, and manuscript revision. MJ was involved in statistical analysis of data and manuscript revision. PK was involved in study design and manuscript preparation and revision. ED was involved in study design and manuscript preparation and revision. BV was involved in overall study design and supervision, data analysis, and manuscript preparation and revision. All authors read and approved the final manuscript.

## Supplementary Material

Additional file 1 **Additional tables**. Table S1: Changes in individual patients' serum vitamin D, parathormone, calcium (total and ionised), creatinine and albumin concentrations at various time points with corresponding values for fluid balance. Table S2: Changes in patients' creatinine, albumin, fluid balance and weight values at various time points. Click here for file
