# Supplementary material for: Acute fluid shifts influence the assessment of serum vitamin D status in critically ill patients
Source: Crit Care. 2010 Nov 26;14(6):R216. doi: 10.1186/cc9341 (PMC3219984; doi:10.1186/cc9341)
Supplement: Additional file 1 — Additional tables. Table S1: Changes in individual patients' serum vitamin D, parathormone, calcium (total and ionised), creatinine and albumin concentrations at various time points with corresponding values for fluid balance. Table S2: Changes in patients' creatinine, albumin, fluid balance and weight values at various time points. [file cc9341-S1.DOC]

| **Patients** | **25 (OH) D_3_**  **nMol/L** | | | | | **1α, 25 (OH)_2_ D_3_**  **pMol/L** | | | | | **PTH**  **pMol/L** | | | | | **Calcium total**  **(ionised) mmol/L** | | | | |
| --- | --- | --- | --- | --- | --- | --- | --- | --- | --- | --- | --- | --- | --- | --- | --- | --- | --- | --- | --- | --- |
|  | **1** | **2** | **3** | **4** | **5** | **1** | **2** | **3** | **4** | **5** | **1** | **2** | **3** | **4** | **5** | **1** | **2** | **3** | **4** | **5** |
| 1 | 51 | 35 | 46 | 51 | 58 | 133 | 72 | 52 | 100 | 180 | 20 | 42 | 12 | 9 | 4 | 2.22 (1.1) | 1.94 (0.84) | 2.02 (1.0) | 2.04 (1.06) | 2.34 (1.15) |
| 2 | 69 | 43 | 62 | 74 | 66 | 150 | 80 | 92 | 156 | 340 | 62 | 18 | 12 | 9.20 | 5.3 | 2.34 (1.16) | 2.0 (0.97) | 2.29 (1.13) | 2.23 (1.20) | 2.246 (1.18) |
| 3 | 23 | 9 | 20 | 24 | 18 | 56 | 25 | 53 | 36 | 89 | 29 | 69 | 14 | 6.30 | 10 | 2.35 (1.09) | 1.84 (0.82) | 2.39 (1.12) | 2.24 (1.17) | 2.32 (1.08) |
| 4 | 45 | 28 | 33 | 39 | 57 | 83 | 40 | 71 | 99 | 199 | 2 | 28 | 5.20 | 12 | 2.9 | 2.18 (1.12) | 1.96 (0.88) | 2.05 (1.03) | 2.0 (1.01) | 2.27 (1.14) |
| 5 | 63 | 35 | 50 | 64 | 85 | 92 | 33 | 89 | 71 | 288 | 24 | 33 | 20 | 16 | 8.1 | 2.26 (1.05) | 1.97 (0.84) | 1.90 (0.97) | 2.06 (1.03) | 2.39 (1.17) |
| 6 | 79 | 49 | 71 | 92 | 87 | 144 | 49 | 102 | 124 | 309 | 7.60 | 68 | 2.9 | 14 | 1.7 | 2.23 (1.2) | 2.04 (0.97) | 2.32 (1.21) | 2.20 (1.14) | 2.44 (1.24) |
| 7 | 50 | 30 | 37 | 38 | 48 | 78 | 24 | 50 | 45 | 246 | 9 | 90 | 9.5 | 9.1 | 2 | 2.24 (1.1) | 1.87 (0.85) | 1.97 (0.98) | 2.14 (1.11) | 2.35 (1.14) |
| 8 | 48 | 28 | 36 | 41 | 49 | 67 | 38 | 62 | 60 | 172 | 16 | 7.6 | 6.2 | 4.3 | 7.1 | 2.18 (0.86) | 1.85 (1.11) | 2.15 (1.17) | 2.11 (1.15) | 2.25 (1.15) |
| 9 | 68 | 47 | 59 | 62 | 63 | 100 | 61 | 156 | 157 | 218 | 11 | 42 | 14 | 13 | 0.50 | 2.37 (1.14) | 2.13 (0.94) | 2.06 (1.10) | 2.10 (1.09) | 2.35 (1.15) |
| 10 | 53 | 23 | 61 | 47 | N/A | 138 | 62 | 85 | 79 | N/A | 22 | 67 | 13 | 22 | N/A | 2.20 (1.18) | 1.82 (0.88) | 1.96 (1.06) | 1.95 (1.09) | 2.40 (1.12) |
| 11 | 50 | 50 | 65 | 30 | 42 | 67 | 63 | 48 | 85 | 110 | 23 | 55 | 18 | 18 | 5.20 | 2.15 (1.07) | 2.02 (0.90) | 2.03 (1.01) | 1.99 (1.08) | 2.33 (1.10) |
| 12 | 76 | 49 | 70 | 78 | 110 | 99 | 59 | 100 | 98 | 280 | 5.2 | 39 | 9.90 | 17 | 7.3 | 2.19 (1.10) | 1.87 (0.77) | 2.0 (0.99) | 1.86 (1.02) | 2.18 (1.09) |
| 13 | 81 | 64 | 46 | 73 | 87 | 80 | 72 | 57 | 97 | 373 | 13 | 5 | 45 | 10 | 2.7 | 2.13 (1.13) | 1.89 (0.89) | 1.91 (0.86) | 1.94 (1.03) | 2.23 (1.18) |
| 14 | 63 | 41 | 58 | 57 | N/A | 106 | 66 | 76 | 114 | N/A | 82 | 24 | 5.2 | 8 | N/A | 2.04 (1.03) | 1.84 (0.88) | 1.81 (0.94) | 1.84 (0.97) | 2.27 (1.02) |
| 15 | 57 | 26 | 41 | 52 | 45 | 65 | 36 | 37 | 65 | 155 | 14 | 25 | 1.8 | 4.1 | 3.7 | 2.11 (1.15) | 1.86 (0.79) | 2.27 (1.15) | 2.23 (1.17) | 2.37 (1.15) |
| 16 | 35 | 22 | 28 | 28 | 36 | 51 | 29 | 43 | 47 | 132 | 11 | 73 | 20 | 15 | 10 | 2.35 (1.16) | 2.05 (0.89) | 2.14 (1.10) | 2.15 (1.12) | 2.38 (1.17) |
| 17 | 83 | 60 | 55 | 63 | 74 | 117 | 91 | 57 | 68 | 94 | 23 | 24 | 20 | 17 | 8.7 | 2.29 (1.05) | 2.08 (0.94) | 2.27 (1.05) | 2.24 (1.09) | 2.33 (1.07) |
| 18 | 76 | 46 | 62 | 69 | 83 | 201 | 93 | 126 | 154 | 327 | 5.2 | 6.50 | 4.4 | 6.50 | 1.80 | 2.24 (1.19) | 1.98 (0.92) | 2.24 (1.15) | 2.08 (1.11) | 2.21 (1.20) |
| 19 | 60 | 29 | 41 | 63 | 67 | 55 | 37 | 50 | 67 | 138 | 23 | 54 | 12 | 12 | 11 | 2.25 (1.16) | 1.93 (0.83) | 2.22 (1.11) | 2.19 (1.10) | 2.49 (1.15) |

**Table showing changes in individual patients’ serum vitamin D, parathormone, calcium (total and ionised), creatinine and albumin concentrations at various time points with corresponding values for fluid balance.**

Table showing changes in patients’ creatinine, albumin, fluid balance and weight values at various time points

| **Patients** | **Creatinine**  **µMol/L** | | | | | **Albumin**  **g/L** | | | | | **Relative fluid balance**  **Litres** | | | | | **Weight**  **Kg** | | | | |
| --- | --- | --- | --- | --- | --- | --- | --- | --- | --- | --- | --- | --- | --- | --- | --- | --- | --- | --- | --- | --- |
|  | **1** | **2** | **3** | **4** | **5** | **1** | **2** | **3** | **4** | **5** | **1** | **2** | **3** | **4** | **5** | **1** | **2** | **3** | **4** | **5** |
| 1 | 77 | 70 | 83 | 76 | 74 | 33 | 24 | 30 | 32 | 31 | 0 | 2.8 | 2.8 | 1.3 | 0.7 | 112.1 | N/A | N/A | N/A | 108.90 |
| 2 | 84 | 73 | 80 | 92 | 92 | 32 | 24 | 35 | 39 | 32 | 0 | 5 | 4.9 | 3.45 | 1.4 | 70.95 | N/A | N/A | N/A | 69.80 |
| 3 | 80 | 74 | 126 | 74 | 74 | 27 | 15 | 26 | 32 | 27 | 0 | 2.5 | 1.15 | 1.80 | 0.75 | 52.30 | N/A | N/A | N/A | 53.90 |
| 4 | 73 | 73 | 89 | 59 | 67 | 36 | 24 | 29 | 31 | 38 | 0 | 2 | 2.15 | 2.85 | 0.85 | 83 | N/A | N/A | N/A | 81 |
| 5 | 78 | 78 | 87 | 80 | 79 | 26 | 16 | 29 | 27 | 31 | 0 | 5.3 | 1.15 | 3.05 | 2.35 | 65 | N/A | N/A | N/A | 67.4 |
| 6 | 65 | 69 | 85 | 77 | 77 | 37 | 24 | 29 | 33 | 34 | 0 | 2 | 3.5 | 1.55 | 0.10 | 109.2 | N/A | N/A | N/A | 108.5 |
| 7 | 62 | 62 | 67 | 61 | 66 | 34 | 24 | 27 | 31 | 32 | 0 | 2 | 4.45 | 2.45 | 2.50 | 68.5 | N/A | N/A | N/A | 68.1 |
| 8 | 74 | 46 | 55 | 84 | 68 | 32 | 23 | 34 | 36 | 34 | 0 | 3.10 | 3.10 | 2.40 | 1.60 | 73 | N/A | N/A | N/A | 71.90 |
| 9 | 83 | 67 | 71 | 68 | 67 | 37 | 31 | 34 | 34 | 34 | 0 | 2.7 | 1.90 | 1.20 | 0.30 | 115.8 | N/A | N/A | N/A | 118.8 |
| 10 | 77 | 59 | 69 | 95 | 89 | 38 | 23 | 36 | 38 | 32 | 0 | 4.50 | 3.65 | 3.25 | 0.30 | 92 | N/A | N/A | N/A | 89.65 |
| 11 | 40 | 52 | 60 | 58 | 54 | 31 | 23 | 27 | 29 | 32 | 0 | 3.80 | 0.50 | 1.60 | 0.10 | 121.95 | N/A | N/A | N/A | 118.45 |
| 12 | 84 | 69 | 89 | 101 | 106 | 34 | 24 | 31 | 34 | 32 | 0 | 4 | 5.6 | 4.85 | 1.65 | 99 | N/A | N/A | N/A | 100.50 |
| 13 | 85 | 85 | 83 | 73 | 81 | 37 | 32 | 27 | 35 | 35 | 0 | 3.60 | 2.75 | 1.40 | 0.20 | 69.55 | N/A | N/A | N/A | 67.70 |
| 14 | 72 | 58 | 74 | 63 | 62 | 31 | 24 | 31 | 32 | 32 | 0 | 2.8 | 3.6 | N/A | N/A | 91 | N/A | N/A | N/A | 85.4 |
| 15 | 59 | 51 | 59 | 67 | 61 | 36 | 22 | 31 | 32 | 35 | 0 | 3 | 3.2 | 2.60 | 1.65 | 62.3 | N/A | N/A | N/A | 60.55 |
| 16 | 90 | 73 | 85 | 105 | 122 | 36 | 25 | 30 | 28 | 34 | 0 | 4.20 | 5.10 | 4.30 | 1.90 | 91.50 | N/A | N/A | N/A | 92.95 |
| 17 | 121 | 109 | 227 | 231 | 240 | 29 | 24 | 19 | 23 | 22 | 0 | 4.40 | 3.80 | 1.30 | 0.90 | 81.20 | N/A | N/A | N/A | N/A |
| 18 | 85 | 73 | 92 | 97 | 74 | 41 | 29 | 32 | 33 | 32 | 0 | 3.80 | 3.40 | 2.10 | 0.60 | 117 | N/A | N/A | N/A | 113.60 |
| 19 | 144 | 101 | 110 | 149 | 151 | 32 | 20 | 28 | 30 | 31 | 0 | 5.10 | 2.40 | 0.75 | 1.10 | 97.50 | N/A | N/A | N/A | 96.40 |
